# A One Health call to re examine medical practice: going beyond strong anthropocentrism

**DOI:** 10.1016/j.soh.2025.100129

**Published:** 2025-10-13

**Authors:** Moshe Porat Wojakowski, Shir Porat-Butman

**Affiliations:** a Oral Medicine Unit, Sheba Medical Center, Tel Hashomer, 5265601, Israel; b “Bina” Program, Faculty of Dental Medicine, Hebrew University of Jerusalem, Jerusalem, 9112001, Israel; c Faculty of Education, Bar-Ilan University, Ramat Gan, 5290002, Israel

**Keywords:** One Health, Public health, Medical ethics, Anthropocentrism, Green oncology

## Abstract

One Health discourse rightly stresses human–animal–environment interdependence, yet its normative footing can be thin and often defaults to strong anthropocentrism, valuing non-human beings and ecosystems only instrumentally. This perspective focuses on clinical decision-making and proposes a shift from strong anthropocentrism to weak anthropocentrism. weak anthropocentrism maintains the human-centered mandate that underpins medicine and public health while at the same time requiring proportionality, least harm, and intergenerational responsibility that acknowledge intrinsic—not merely instrumental—value in ecological systems and non-human life. Building on One Health's systems thinking, the framework offers a pragmatic approach rather than a radical turn to biocentrism or ecocentrism, and it recognizes ongoing debates about prudential versus radical interpretations within One Health. We illustrate feasibility with green oncology, where clinically equivalent options are selected to reduce life-cycle emissions, waste, and antimicrobial pressures—showing how ecological considerations can be integrated without compromising patient or population outcomes. The proposal aligns with contemporary regulatory strategies that recommend a One Health lens and invites a practical recalibration of public health policy: expand evaluative criteria, embed ecological reasoning in decision processes, and align clinical benefit with long-term planetary stewardship. Weak anthropocentrism thus offers a workable, ethically coherent route to extend “do no harm” beyond humans while remaining faithful to the core commitments of both clinical care and public health.

## Graphical abstract

**Figure:**
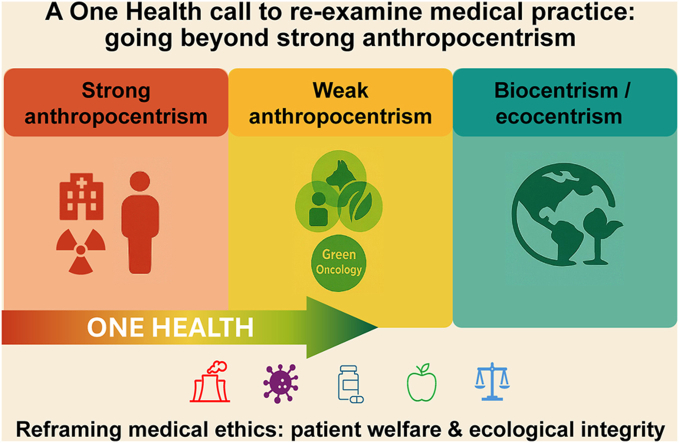

## 1 Introduction

Modern medicine is explicitly devoted to human health and welfare (Declaration of Geneva) [1]. It is reflected in a clinical paradigm that evaluates treatments and public health measures primarily by their consequences for individuals. For example, waste absorbed by ecosystems or animals culled to control disease are usually noted only when they threaten human well-being. Laboratory science, long anchored in animal models, has only recently begun shifting toward more human-relevant and humane alternatives [2]. In nearly all cases, environmental or animal impacts are addressed insofar as they feed back to human health, not as independent moral concerns.

Even environmental damage is typically noted only when it harms humans—through climate change, air pollution, antimicrobial resistance (AMR), or food insecurity—rather than because non-human beings or ecosystems possess intrinsic value [[3], [4], [5]]. This anthropocentric reflex has cultural roots: as Lynn White argued, Western traditions cast humans as lords over creation (Genesis) [6]. Even when medicine broadened to Engel's biopsychosocial framework—recognizing mind and society in illness and healing—the evaluative center remained human [7,8].

One Health was conceived to operationalize the interdependence of human, animal, and ecosystem health. The One Health High-Level Expert Panel (OHHLEP) explicitly states: “It recognizes that the health of humans, domestic and wild animals, plants, and the wider environment (including ecosystems) are closely linked and interdependent” [9]. This parallels holistic approaches in many indigenous knowledge systems [2]. Yet, despite this rhetoric, practical implementations in medicine often revolve around safeguarding humans; animals and ecosystems are treated as vectors, reservoirs, or protective buffers rather than as entities with intrinsic worth [9]. The deeper ethical question—whether harming non-human beings or degrading ecosystems is wrong in itself—remains unsettled. In public-health discourse, environmental harms are typically framed as externalities to be internalized because they threaten people, not because duties are owed to the non-human world [3,4].

This paper seeks to: (1) address the gaps in One Health by introducing a moral–theoretical framework; (2) illustrate, through the example of green oncology, how weak anthropocentrism can shift clinical practice from isolated sustainability efforts to more coherent, system-aware decisions; and (3) explore the anthropocentric foundations of modern medicine to explain why moving from strong to weak anthropocentrism is both necessary and feasible. Building on Norton's distinction between strong and weak anthropocentrism [10], alongside adjacent bio- and eco-centric views, we advance weak anthropocentrism as a pragmatic ethic for One Health. Weak anthropocentrism keeps patient welfare primary while recognizing intrinsic value in non-human life and ecological integrity, supplying the ethical compass One Health lacks. Green oncology illustrates how weak anthropocentrism moves practice from ad hoc sustainability fixes to coherent, system-aware decisions. The next section traces the anthropocentric roots of the medical paradigm and motivates a shift from strong to weak anthropocentrism.

## 2 The medical paradigm and its anthropocentric foundations

In the late 19th and 20th centuries, classical biomedicine consolidated disease as a biological defect and cure as its technological correction [7,11]. Health was reduced to molecular and organ-level dysfunctions, while nature was cast as resource or threat. Environmental costs of care—energy, chemical waste, emissions—were ignored because the evaluative horizon ended at the patient's body and short-term welfare, reflecting epistemic and moral reductionism. Engel's 1977 manifesto broadened the paradigm by linking illness to psychological and social factors and situating patients within families, culture, and socioeconomic contexts [7,8]. Yet the biopsychosocial model remained anthropocentric: ecosystems and non-human life counted only insofar as they affected human well-being [12,13]. Knowledge expanded; the human-centered moral boundary stayed put.

Assessing whether medicine can widen its moral horizon requires precise terms. Norton's typology supplies them [10,14]. Strong anthropocentrism holds that only human interests possess intrinsic value; environmental harms matter only via human risk. Weak anthropocentrism retains the human vantage point but allows intrinsic value to be ascribed to non-human beings and systems on cultural, aesthetic, scientific, or moral grounds. A thought experiment illustrates the difference: in the “Last Man” scenario, the final human on Earth contemplates destroying a thriving ecosystem. Here, strong anthropocentrism finds no wrong, whereas weak anthropocentrism condemns the destruction for violating ecological integrity [15]. Beyond weak anthropocentrism lie biocentrism, which values all living organisms [16], and ecocentrism, which extends standing to ecosystems and processes [17,18]. These horizon points clarify how weak anthropocentrism departs from strong anthropocentrism without abandoning patient focus.

Although One Health links human, animal, and ecosystem health [19], the current practice often remains strongly anthropocentric. For example, culling, farming, and habitat protection are typically justified by how they protect human communities [12,20,21]. Without an explicit path from strong anthropocentrism to weak anthropocentrism, One Health risks becoming a technocratic variant of the very paradigm it aims to transcend.

Weak anthropocentrism offers that bridge. This ethical gap underscores that if non-human life has value, it must enter clinical reasoning [10]. Embedding weak anthropocentrism would shift animal and environmental protections from tactics to duties. It will require assessing harms such as culling or intensive farming in their own right, and expanding governance indicators to include ecological integrity [12,19,22]. The shift widens the circle of intrinsic concern while preserving a privileged—though not exclusive—status for human welfare. Operationally, decisions proceed by: (1) clarifying human goods—efficacy, safety, quality of life, and equity; (2) simultaneously assessing ecological impacts; and (3) applying proportionality—prefer functionally equivalent options with lower ecological harm, or else justify, mitigate, and monitor harms via ecological indicators. This reframes practice from narrow human risk management (strong anthropocentrism) to minimizing harms within human–ecological systems (weak anthropocentrism), without requiring full biocentrism.

## 3 One Health and the need for an ethical compass

To avoid reproducing strong anthropocentrism under a systems thinking vocabulary, the One Health agenda must be read through the ethical axis developed above. Within the One Health literature, a distinction is drawn between a “Prudential” and a “Radical” One Health agenda [23]. In brief, “Prudential” One Health agenda retains a human-centered, policy-tractable orientation with ecological measures largely instrumental. In contrast, the “Radical” One Health agenda calls for a non-anthropocentric turn that grounds duties to ecosystems and widens moral standing. Accordingly, and read alongside the Prudential/Radical distinction, we organized our analysis according to the six official action tracks of the Quadripartite *One Health Joint Plan of Action (2022–2026):* (1) strengthening One Health capacities and governance; (2) emerging and re-emerging zoonotic epidemics and pandemics; (3) endemic zoonotic, neglected tropical, and vector-borne diseases; (4) food safety; (5) AMR; and (6) environment, climate change, and ecosystem health [22]. Together they capture the principal interfaces where medical and public-health practice touch non-human life and ecological processes.

Table 1 maps each domain of the six official action tracks of the *One Health Joint Plan of Action* *(2022–2026)* along the strong anthropocentrism, weak anthropocentrism, biocentrism/ecocentrism axis, showing how justificatory logic, decision rules and metrics shift once intrinsic ecological value is acknowledged.

**Table 1:** Ethical orientations across One Health domains.

One Health Joint Plan of Action track	Strong anthropocentrism	Weak anthropocentrism	Biocentrism/ecocentrism	Implications for medical practice
Strengthening One Health capacities and governance	Multi-sectoral coordination focused on human threats; dashboards track human-relevant risks	Mandates include animal and ecosystem health as ends in themselves; monitor habitat loss and species decline as triggers	Non-human stakeholders gain standing; policies may be vetoed for ecosystem harm	Establish cross-sector committees including ecological expertise; integrate environmental indicators into health-system metrics
Emerging and re-emerging zoonotic epidemics and pandemics	Wildlife/livestock monitored or culled to protect humans; focus on human morbidity and economy	Habitat conservation and animal welfare weighed as duties; culling only after proportionality tests include non-human harms	Ecosystem integrity and species welfare constrain interventions; mass culling requires exceptional justification	Create ethical review panels with animal/ecology expertise; integrate habitat metrics into outbreak-preparedness plans
Endemic zoonotic, neglected tropical, and vector-borne diseases	Human-centric eradication campaigns with little attention to wildlife reservoirs or ecological spillovers	Extend duties to protect non-human hosts and their habitats; evaluate ecological side-effects of control programs	Biodiversity preservation treated as a moral duty; disruption of ecological balance condemned regardless of human impact	Integrate ecosystem-health indicators into surveillance and disease-control programs; promote habitat restoration as prevention
Food safety	Optimize human nutrition and market stability; animal welfare secondary to productivity	Agro-ecosystem health and animal welfare gain intrinsic weight; deforestation and biodiversity loss condemned beyond human costs	Agriculture governed by ecosystem carrying capacity and species integrity	Include sustainability and welfare criteria in dietary guidelines and procurement policies; prefer agroecological models
AMR	Stewardship to preserve drug efficacy for patients; farm limits to block human infection pathways	Limit environmental antibiotic loads (hospital effluent and pharma waste) because microbial ecologies matter morally	Microbiome resilience (soil, water, and animal hosts) becomes a target; intensive farming prophylaxis ethically questionable	Add environmental antibiotic indicators to stewardship KPIs; redesign waste streams and procurement
Environment, climate change, and ecosystem health	Environmental degradation noted mainly when it harms humans (pollution, vector spread, and extreme heat)	Ecological systems recognized as intrinsic goods; mitigation guided by proportionality and least harm to habitats and future generations	Ecosystem integrity and planetary boundaries treated as moral constraints on action	Pair clinical outcomes with CO_2_e, water, and biodiversity metrics; embed “do no significant ecological harm” clauses in health planning and infrastructure decisions

Note: The table aligns with the six official *One Health Joint Plan of Action* (2022–2026) tracks: (1) strengthening One Health capacities and governance; (2) emerging and re-emerging zoonotic epidemics and pandemics; (3) endemic zoonotic, neglected tropical and vector-borne diseases; (4) food safety; (5) antimicrobial resistance; and (6) environment, climate change and ecosystem health. It illustrates how justificatory logic, decision rules, and ethical duties evolve along the strong-anthropocentrism → weak-anthropocentrism → biocentrism/ecocentrism axis. Equity and justice are treated as cross-cutting ethical principles embedded across all tracks. Abbreviations: AMR, antimicrobial resistance; KPI, key performance indicators; LCA, life-cycle assessment; CO_2_e, carbon dioxide equivalents.

Under weak anthropocentrism, we do not shortcut ecological appraisal: the same harms to habitats, species, or microbial ecologies that a biocentric/ecocentric position would flag must be identified and weighed. AMR illustrates the point. One need not speak of “rights” for bacteria to consider moral salience to microbial communities. However, their integrity underpins ecosystem functioning and future therapeutic options. Weak anthropocentrism therefore treats environmental antibiotic loads and wastewater effluents as wrongs to be minimized—not only because they rebound on humans, but because degrading microbial ecologies is itself a harm that demands justification.

Read this way, One Health becomes more than technocratic coordination: it acquires an explicit ethical compass relevant for the pragmatic application by medical practitioners, especially when this type of guidance is lacking in existing approaches. The next section shows how this compass can guide concrete clinical decisions by examining green oncology as a worked example.

## 4 Case study: green oncology as a model for weak anthropocentrism

Oncology concentrates nearly all One Health action tracks. Cancer care entails energy-intensive imaging and radiotherapy (environment–climate); intensive antibiotic use for neutropenia and prophylaxis (AMR); complex pharmaceutical supply chains with cytotoxic-waste streams (governance/effluent control); research ethics structured by the “3Rs”—replacement, reduction, refinement—first articulated by Russell and Burch [24]. Originally intended to replace animal methods where feasible, reduce animal numbers, and refine welfare, the 3Rs now underpin regulatory expectations [24,25]. For instance across preclinical oncology, early-phase screening can replace certain animal assays with human tumor organoids; rigorous power calculations and shared-control designs reduce animal numbers; and humane endpoints with minimally invasive sampling refine tumor-bearing models—operationalizing Russell & Burch's 3Rs and the requirements of the EU Directive [24,25]. Under strong anthropocentrism, these impacts are ethically mute so long as patient benefit is maximized. Much of the “green oncology” literature reflects this reflex: the Italian *Collegio Italiano dei Primari Oncologi Medici Ospedalieri* (CIPOMO) “Green Oncology Position Paper” and its follow-up [26] advocate fewer disposables, oral regimens, and biosimilars largely because such changes save money and, ultimately, protect human health systems. Epstein and colleagues' call to move from a “gold” to a “green” era [27] goes further rhetorically, but still stops short of an explicit claim that ecosystems have standing in their own right.

A weak anthropocentric reframing alters the justification, not necessarily the choice of therapy: identical clinical nodes are re-evaluated through proportionality and least-harm to coupled human–ecological systems. If two imaging modalities are clinically equivalent, weak anthropocentric obliges the lower-emission option because ecological integrity matters intrinsically. For example, antibiotic policies are assessed not only for resistance risk to future patients but also for damage to microbial ecologies downstream of hospital effluent [28,29]. Moreover, waste routes and procurement are reviewed with interspecies and intergenerational justice in view, consistent with One Health governance principles [22]. Drug development can continue to adhere to the “3Rs”. In short: “all necessary means for this patient, constrained by duties to ecological systems.” The analytic sequence used in this paper—mapping strong anthropocentric defaults, applying weak anthropocentric constraints, flagging biocentrism/ecocentrism boundaries—can be generalized to other specialties; oncology merely makes the interdependence visible.

## 5 Conclusions and future directions

Medicine's paradigms remain anchored in strong anthropocentrism: environmental and animal harms count ethically only when they boomerang on humans. One Health offers a systems thinking scaffold but, absent of an explicit moral compass or a practical approach for medical practitioners, often reproduces this stance. Drawing on Norton, we proposed weak anthropocentrism as a pragmatic midpoint: patient welfare stays primary, yet non-human beings and ecological systems possess intrinsic value that must be weighed through proportionality and least-harm reasoning. Mapping the Quadripartite One Health action tracks along the strong anthropocentrism–weak anthropocentrism–biocentrism/ecocentrism axis shows where justificatory logic, decision rules and metrics must shift. Oncology illustrates operationalization: identical clinical benefits should default to lower ecological harm; antimicrobial and waste policies must consider microbial and ecosystem integrity, not merely future human risk. Adopting weak anthropocentrism does not solve every dilemma, but it supplies the missing ethical compass for genuinely sustainable, justice-attentive public-health and clinical practice. This is particularly important for medical practice currently lacking this type of guidance.

## CRediT authorship contribution statement

**Moshe Porat Wojakowski:** Writing – review & editing, Writing – original draft, Project administration, Methodology, Investigation, Formal analysis, Data curation, Conceptualization. **Shir Porat-Butman:** Writing – review & editing, Validation, Resources, Investigation, Formal analysis.

## Declaration of competing interests

The authors declare that they have no known competing financial interests or personal relationships that could have appeared to influence the work reported in this paper.
